# Maternal adaptation to caring for a child with congenital Zika virus syndrome

**DOI:** 10.1590/0034-7167-2025-0475

**Published:** 2026-10-16

**Authors:** Laís Celeste Medeiros Mendes da Fonseca, Fernanda Machado Silva-Rodrigues, Natália Maria Vieira Pereira-Caldeira, Aline Cristiane Cavicchioli Okido, Lucila Castanheira Nascimento, Diene Monique Carlos, Flávia Azevedo Gomes-Sponholz, Nayara Gonçalves Barbosa

**Affiliations:** I Universidade Federal de Juiz de Fora. Juiz de Fora, Minas Gerais, Brazil; II Universidade de São Paulo, Escola de Enfermagem. São Paulo, São Paulo, Brazil; III Universidade de São Paulo, Escola de Enfermagem de Ribeirão Preto. Ribeirão Preto, São Paulo, Brazil

**Keywords:** Infectious Disease Transmission, Vertical, Zika Virus Infection, Mother-Child Relations, Coping Skills, Nursing., Transmisión Vertical de Enfermedad Infecciosa, Infección por el Virus Zika, Relaciones Madre-Hijo, Habilidades de Afrontamiento, Enfermería.

## Abstract

**Objective:**

to understand the process of maternal adaptation to caring for a child with Congenital Zika Virus Syndrome.

**Methods:**

a descriptive, qualitative study grounded in Roy’s Adaptation Model. Twenty mothers who care for children with this syndrome, from different Brazilian states, were interviewed remotely between January and October 2024. The data were subjected to reflexive thematic analysis.

**Results:**

The mothers experienced challenges related to the diagnosis, the grief for the idealized child, and the adaptation to a new reality. The adaptive modes highlighted physical and emotional overload, the reorganization of self-concept and role performance, and the fragility of support networks.

**Final considerations:**

The findings reinforce the need for professional support and intersectoral strategies that expand assistance to mothers, promoting psychosocial care, social inclusion, and access to specialized services.

## RESUMEN

**Objetivo:**

comprender el proceso de adaptación materna al cuidado del niño con el Síndrome Congénita del virus del Zika.

**Métodos:**

estudio descriptivo y cualitativo, fundamentado en el Modelo de Adaptación de Roy. Se entrevistó de forma remota a 20 madres cuidadoras de niños con este síndrome, provenientes de diferentes estados brasileños, entre enero y octubre de 2024. Los datos fueron sometidos a análisis temático reflexivo.

**Resultados:**

Las madres experimentaron desafíos relacionados con el diagnóstico, el duelo por el hijo idealizado y la adaptación a una nueva realidad. Los modos adaptativos evidenciaron la sobrecarga física y emocional, la reorganización del autoconcepto y del desempeño de roles, además de la fragilidad de las redes de apoyo.

**Consideraciones finales:**

Los resultados refuerzan la necesidad de apoyo profesional y de estrategias intersectoriales que amplíen el soporte a las madres, con el fin de promover asistencia psicosocial, inclusión social y acceso a cuidados especializados.

## INTRODUCTION

Infection with the Zika virus represents an important health concern for pregnant women, as the virus can be transmitted transplacentally and cause teratogenic effects in the fetus. The spectrum of signs and symptoms resulting from these effects has been termed Congenital Zika Virus Syndrome (CZVS), which includes a range of structural brain abnormalities and functional impairments in affected children, secondary to damage to the central nervous system^(1,2)^. CZVS manifests primarily as microcephaly, present in approximately 80% of cases. In addition, various neurological, visual, motor, and auditory complications may occur^(1-3)^. Infection with the Zika virus during the first and second trimesters of pregnancy carries an increased risk of intrauterine growth restriction and fetal central nervous system impairment^(1)^.

The Zika virus epidemic in Latin America between 2015 and 2019 led to the birth of children with CZVS^(4)^. Brazil was the most affected country^(5)^, accounting for about 80% of cases, and it continues to report new infections, highlighting an ongoing threat to maternal and child health. Vulnerable populations are disproportionately affected, as they often face precarious socioeconomic conditions, low educational levels, inadequate housing, poor sanitation, and high exposure to disease vectors^(3,6)^. These groups also have reduced access to reproductive health services, such as prenatal care and contraceptive counseling, which deepens existing health inequities^(1)^. Social inequalities and poverty are even more pronounced among women who assume primary responsibility for caring for affected children^(3)^.

The social, economic, cultural, and personal repercussions of this epidemic remain insufficiently understood, and its magnitude poses challenges for health systems and professional training^(1)^. The consequences of CZVS extend beyond the affected children to their families, communities, and caregivers - especially mothers, who are more prone to mental distress due to the complexity of the required care^(2)^. Children with CZVS may present physical or cognitive functional limitations, dependence on medications, specialized diets, or assistive technologies, and require rehabilitation therapies and multidisciplinary care^(7)^. These characteristics place them within the group of children with special health care needs, who require more intensive monitoring and support from health services compared to other children of the same age group^(8)^.

Caring for children with Congenital Zika Virus Syndrome (CZVS) places mothers in situations marked by concrete challenges, emotional dilemmas, and profound life changes. The daily experience often involves reconfiguring life projects, facing physical, psychological, and financial overload, navigating barriers to health services, and dealing with uncertainty about the child’s future and development. These experiences are accompanied by emotions such as guilt, fear, sadness, loneliness, uncertainty, stress, anxiety, adaptation, stigma, and social prejudice related to the child’s condition^(3,5,7)^. Many mothers experience distancing from social, cultural, and leisure activities, along with fragile support networks and the need to share caregiving responsibilities^(6,7)^, with repercussions to their health and well-being^(1)^.

The caregiving context exposes them to multiple stimuli that interfere with their ability to adapt to the new situation imposed by CZVS. Given the substantial limitations imposed by the syndrome and the meanings attributed to the child’s condition, understanding maternal adaptation becomes essential. Callista Roy’s theoretical model offers a valuable framework for this purpose. In this model: The person is viewed as a holistic adaptive system capable of generating either adaptive or ineffective responses; The environment encompasses all conditions, circumstances, and influences that shape the person’s development and behavior^(9,10)^; In this perspective, the implemented nursing care aims the promotion of adaptative responses from the person^(9,10)^.

Deepening knowledge on this topic can support the development of strategies to better assist mothers and families caring for children with CZVS, by considering the path they navigate in response to life stimuli - that is, the consequences of the syndrome and their involvement as primary caregivers - as they strive to strengthen their adaptive capacity^(9)^.

## OBJECTIVE

Understanding the process of maternal adaptation to caring for a child with Congenital Zika Virus Syndrome.

## METHODS

### Ethical aspects

The study was conducted in accordance with national and international ethical guidelines and was approved by a Research Ethics Committee, part of the CEP/Conep system, on September 30, 2022. To preserve participant anonymity, the interviews were identified using an alphanumeric code consisting of the letter “P” (participant) followed by an Arabic numeral corresponding to the chronological order in which the interviews were conducted (P1 to P20).

### Type of study

Qualitative study grounded in Roy’s Adaptation Model (RAM). According to Roy, individuals exist in constant interaction with their environment and are continuously exposed to stimuli, which she defines as *inputs*. These stimuli are classified as focal (internal or external), contextual, and residual. The combination of these stimuli determines the individual’s level of adaptation, meaning their capacity to confront and respond to environmental changes. The response to these stimuli - referred to as *output* - may be an effective adaptive response or an ineffective maladaptive response^(9,10)^.

Roy’s Adaptation Model (RAM) is composed of four adaptive modes that are produced or manifested. In the physiological mode, the individual reacts to stimuli from the environment in relation to five basic needs: oxygenation, nutrition, elimination, activity, and rest. The self-concept mode encompasses psychological, moral, and spiritual aspects, along with the individual’s values, beliefs, and feelings. The role function mode is social in nature and relates to the roles the individual assumes in society. Finally, the interdependence mode, also social in nature, refers to interactions involving giving and receiving love, respect, and value^(9,10)^.

Given the complexity involved in the adaptation of mothers of children affected by CZVS, it is essential to analyze which elements were present in their coping mechanisms when faced with the new stimulus of being the mother of a child with CZVS, which adaptive modes they developed in this process, and what behavioral outcomes (adaptation or maladaptation) they presented in response to this new life condition. The study followed the Consolidated Criteria for Reporting Qualitative Research (COREQ)^(11)^.

### Setting, Population, and Selection Criteria

Biological mothers of children diagnosed with Congenital Zika Virus Syndrome, aged 18 years or older, and located through social media were included. Biological mothers who were not responsible for the child’s care - when caregiving was performed by another responsible caregiver - and mothers of adolescents were excluded.

### Data collection

Data were collected between January and October 2024. Recruitment of potential study participants was carried out through dissemination of the research on social media, specifically Instagram® and Facebook®. To locate potentially eligible profiles, the following search terms were used: Zika virus, Zika and pregnancy, and congenital Zika virus syndrome. Searches were performed manually using the platforms’ internal search tools to identify posts, profiles, pages of non-governmental organizations (NGOs) that support families of children with CZVS, and groups whose content related to the study topic. A search was also conducted through the followers list of these groups or pages, with special attention to users who interacted with the posts.

Open profiles on social media were prioritized, as they often included in their descriptions a reference to a child with CZVS, as well as publicly shared photos of the child. Once the profiles were identified (n = 168), an individual invitation was sent via direct message to the participants’ social media accounts. This message included an introduction of the principal researcher, the study objectives, and an email address for contact, with the purpose of clarifying questions and formalizing interest in participation.

The sampling technique used was snowball sampling^(12)^, which consists of asking the initial participants to refer other people with characteristics similar to those desired for the study. Thus, in addition to direct invitations, some participants suggested other mothers who might be interested in contributing to the research, expanding the diversity of data relevant to the study.

The Free and Informed Consent Form was made available for reading. After agreeing, the potential participants were contacted again to schedule the semi-structured interviews, which were conducted remotely and recorded using the Google Meet® platform. The interviews were conducted by a nurse (initials omitted for blinded peer review), a specialist in women’s and children’s health, a master’s student, with previous experience and specific training in conducting qualitative interviews. They lasted an average of 29 minutes and were carried out in a single session. In total, 21 mothers expressed interest in participating, of whom 20 effectively took part in the study. There was one refusal, motivated by the participant’s decision not to share personal matters.

Sociodemographic data were collected, including maternal age, self-reported race, educational level, marital status, and number of children. Regarding the child’s data, age and sequelae of CZVS were obtained. The following guiding questions were used: How did you learn about the diagnosis of congenital Zika virus syndrome? What feelings did you experience at that moment? How did you deal with those feelings? Did anyone support you? What were the consequences of the disease for the child, and what care needs does your child have? Do you have a support network? How did you adapt to the new reality? What is your routine for caring for the child at home?

Participant inclusion was concluded after the 20th interview, when the concurrent analysis of the data indicated the occurrence of theoretical saturation, defined as the point at which the information became repetitive and allowed for deeper exploration of the study questions, anchored in the theoretical model^(12)^. To ensure adherence to this criterion, thematic coding was employed, conducted by two researchers who monitored the recurrence of codes and confirmed the absence of new data capable of significantly contributing to the understanding of the phenomenon under investigation^(12,13)^.

### Data analysis

The data obtained through the interviews were analyzed according to the framework of reflexive thematic analysis, as proposed by Braun and Clarke^(14)^. Based on this type of analysis, the aim was to go beyond the semantic content of the data to examine participants’ ideas, assumptions, and conceptualizations. The six prescribed phases were followed: (1) familiarization with the data through vertical and horizontal readings of the dataset; (2) generation of initial codes based on elements that addressed the study questions; (3) search for themes in which, based on the previous phase, the codes were grouped in light of the theoretical framework; (4) review of the themes; (5) definition and naming of the themes; and (6) production of the report^(14)^.

The analysis was conducted by two researchers (initials omitted for blinded peer review) in order to ensure methodological rigor and reduce preconception biases^(14)^. The codes obtained in the first phase were checked by senior researchers (initials omitted for blinded peer review) and, after validation, organized into themes during meetings among members of the research group. Any disagreements among the authors were resolved in these meetings until consensus was reached.

## RESULTS

Twenty biological mothers of children with Congenital Zika Virus Syndrome participated, with ages ranging from 24 to 45 years. Regarding race, 11 self-identified as mixed race (parda), five as white, and four as Black. The mothers lived in different Brazilian states: six in Ceará, three in Bahia, three in Paraíba, two in Rio Grande do Norte, and one participant in each of the following states: Pernambuco, Goiás, Tocantins, Sergipe, and Rio de Janeiro. Most were married or in a stable union (n = 10), had completed high school or higher education (n = 16), and were dedicated to household activities (n = 19). The Catholic religion was reported by 10 mothers. The number of children ranged from 1 to 4. The age of the children with Congenital Zika Virus Syndrome ranged from 5 to 9 years; all had microcephaly, 11 had cerebral palsy, and 10 had ocular impairment.

Based on the data analysis, three themes were constructed: (i) From grieving the loss of the ideal to adapting to a new reality; (ii) Care demands of the child with Congenital Zika Virus Syndrome: personal and social repercussions for mothers; (iii) Support networks and adaptation strategies. These themes are presented below.

### From grieving the loss of the ideal to adapting to a new reality

This theme describes the coping mechanisms used by mothers when faced with the diagnosis of CZVS. The participants reported that, at first, such behaviors were related to the shock of the new situation, as well as to the performance of new roles, especially that of caregiver to a child with special health needs. The absence of residual stimuli, combined with the novelty of the experience of having a child with a disability, made CZVS - being a new condition in terms of diagnostic and prognostic criteria - render the adaptive process to this reality even more complex, as can be observed below:

*A hole opened beneath my feet because, until then, I already had two children. In my family there wasn’t, there had never been anyone with special needs… There had never been anyone with any problem or malformation. And that was what, for me, was something new, different.* (P7)

*I was left without a reaction, because it is something new. It is a disability in itself, but it is something new. You are not used to, every day, or when you are pregnant, having your child be born with some disability. It was something new, unexpected, that no one imagined. So everyone was very frightened.* (P13)

*I found myself scared, in panic, because I didn’t know exactly what this was that they had told me, nor where it would lead, while at the same time I felt in mourning for not having the child I expected.* [...]. (P6)

Another participant described experiencing grief not only as an isolated event resulting from the loss of the idealized child, but as a continuous process with each new health need of the child. This cycle was perpetuated, in part, by the absence of contextual and residual stimuli that could support other coping mechanisms:

*I lived in mourning for a year, then I began to cope better. I say cope, but with each diagnosis I receive, with each “oh, surgery is needed,” we start pulling ourselves together again. Until a certain age, we had that hope, but as time went by, we understood that no, that it had to be that way and that we needed to be strong. And it was very difficult for us.* (P18)

With the diagnosis of the disease, various demands and care needs of the child emerged - contextual stimuli. At the beginning of the adaptive process, the mothers reported fear and insecurity regarding their ability to care for the child, especially due to the difficulties imposed by the sequelae and consequences of CZVS. In this context, behaviors related to the articulation of mechanisms involving self-concept and role performance emerged as ways of coping with the new reality. Among the challenges mentioned, difficulties with feeding the child, episodes of seizures, and irritability and agitation stood out:

*A then comes the fear, the worry of not knowing how to take care. He is my first and only child. So, besides not really knowing how to care for a newborn in general, there was also the situation of a baby who wasn’t breastfeeding, who wasn’t sucking… It was a child who had seizure episodes, and I didn’t know he was having seizures; he was very irritable and didn’t sleep at night. It was very complicated.* (P2)

*In the beginning, she was a normal baby, who breastfed well until six months* [...]*. As she grew, things started to change. So, what we see is that she began having difficulty drinking liquid milk. Then several tests were done to find out whether she choked on liquid milk or on thick* [pureed] *milk. So, we adapted.* (P13)

### Care demands of the child with Congenital Zika Virus Syndrome: personal and social repercussions for mothers

From the initial adaptive process, marked by the diagnosis of CZVS, the women reported the *self-concept mode* of coping, involving their psychological and spiritual integrity. In this sense, they sought to adapt to their new “self” and to their new realities. They identified, as one of the main care needs, the administration of diet via gastrostomy or the grinding of foods when feeding orally, often due to dysphagia. In their daily care routines, they highlighted medication administration, oxygen therapy, diaper changes, and bathing, all contextualized within solitary caregiving and interdependence:

*I administer the diet. Hydration, medications, diaper changes, bathing, those things.* (P12)

*She has a care routine. You always have to be careful to give the milk at the right time, lay her down, put her in the wheelchair. She has oxygen….* (P20)

[...] *For example, at mealtime, he takes a long time to eat. I prefer to feed him slowly rather than feed him quickly and have him choke or aspirate.* (P2)

*Today she depends exclusively on me. Whether it’s to get a diaper, to eat, to take a bath, to get dressed.* (P13)

The children’s growth and development processes - usually a constant focus of vigilance for most mothers - not only generated expectations regarding their progress over time but also brought increasing concerns and demands. The children’s difficulty in communicating made it harder to understand their specific needs, while typical and expected developmental processes, such as weight gain, resulted in new care demands and additional burden for the mothers:

*Her inability to communicate verbally is a challenge for me. I think that is why I am so intensely focused on her 24 hours a day* [...]*. I am very concerned about her care. This is one of the factors that affects me psychologically the most, you know?* (P10)

*The greatest difficulty today is his weight. Carrying him, moving him from one place to another. It is part of everyday life, and it is becoming increasingly difficult. Because I realize that in three or four years, I will need help just to move him from one place to another* [...]*. They grow up, don’t they?* (P8)

To meet their children’s needs, some mothers had to make significant sacrifices, such as leaving their jobs and professional lives or adapting their work routines to dedicate themselves fully to caregiving. In this sense, in response to the focal and contextual stimuli they faced, it was possible to identify coping modes that affected their social relationships and their previous conditions as autonomous women - an interdependent mode of behavior that made it possible to care for their child:

*A right after he was born, I resigned from my job* [...]*. And I began living to take care of him. And even today I can’t see myself leaving him with someone else and going to work, because it’s very demanding, very rushed.* (P8)

*In fact, my life stopped there. And today I am more the mother, the caregiver, than myself, you know? I wasn’t able to go back to work. So, in a way, I abandoned my life and became only Manoela’s* [fictitious name] *mother and her caregiver.* (P12)

The multiple demands and the need to reconcile the child’s continuous care with other family responsibilities often occurred at the expense of the mothers’ social life, self-care, and overall quality of life.

*Today, regarding my life, taking care of her and being a mother, the feeling I have is one of great love and gratitude. I miss, you know, being myself. I miss that, I won’t deny it.* (P12)

*So, when it comes to going out to a party, going out with friends, I have a lot of difficulty, because I have my family who supports me, but everyone works, so it’s not always that I can leave her with someone.* (P12)

In this context, two participants reported coping strategies to minimize the impact on their self-care caused by the child’s demands: one of them took her daughter to the beauty salon with her, while the other developed her own skills to carry out her self-care. It was therefore possible to observe a role-performance mode intertwined with self-concept as a way of coping and responding to the focal stimulus.

*So I stopped working, I only worked for her father. And today I only take care of Beatriz* [fictitious name]*… Always taking her along to get my nails done, to do my hair with her.* (P19)

*I do my own self-care now, I can do it. I watch videos and I learned to do everything myself, I don’t go to the salon.* (P16)

### Support networks and adaptation strategies

This theme revealed an adaptive process in caring for a child with CZVS, one that seeks a mode of interdependence but within a weakened support network. In this context, the fathers (men), when present, direct their activities toward the family’s subsistence, as the family often faces financial difficulties. For some participants, support from the grandmothers was one of the alternatives they found.

*Only my husband, in this case. When he is at home, because he works.* (P12)

*I never leave him with anyone because I don’t really have anyone. His father never has time off* [...]. *Because it is a matter of having another person to feed; when we plan to have a child, we plan within our budget.* (P7)

*It has always been me and my husband. He has always been my support. It was never anyone else in the family, especially because my family lives in another city, farther away.* (P13)

*Sometimes my mother* [grandmother]*, but since she works, it becomes complicated, you know? So, on weekends, I have the child’s father’s support.* (P15)

There were also reports about the existence of an informal support network, made up of other mothers and families of children also diagnosed with the syndrome, with whom it was possible to exchange information and share experiences, both virtually and in person. This provided, in a certain way, emotional support.

[...] *And later it was really the support chain that was formed through families who were in the same situation. As we got to know each other and placed ourselves in an environment where several people were going through the same thing, it ended up being comforting.* (P2)

*So, one of them* [mother of a child with CZVS] *decided to create a WhatsApp group. And there we got to know each other, we formed a family, and we started sharing information with one another* [...]. (P6)

*I made many friendships; I know almost all the mothers here in the city. Especially those of children with microcephaly or Zika virus syndrome* [...]. *We have a group where we exchange information, one helps the other. For example, I didn’t know how to take legal action for anything. Today I can already do it, I already know where to start.* (P17)

Regarding the adaptations and overcoming of the process, the reports indicate resilience and personal strengthening over time, which characterizes the *self-concept* and *role-function* modes, especially driven by the desire to provide their children with a better quality of life and to ensure their rights.

*I think I learned a lot about seeking things out, about believing in myself. I didn’t think I had the courage I have today, the courage he showed me I have. To leave my city, to restart my life from zero, to go back to studying, to chase things, to fight legally for what belongs to him, to fight with the school, to call the TV to make a scene. Because we are considered “troublemakers” for fighting for my son’s rights. I learned not to fear anything.* (P17)

*I think the desire I have to thrive, to give my son a better quality of life, to be able to give him better care, to be able to go back to work as well. I am trying to give a new meaning to my life.* [...] *Because I don’t want this mourning anymore. I fought my fight.* (P2)

Finally, despite all the repercussions they experienced, the statements reveal that these mothers gradually found balance through different modes of adaptation, expressed by a sense of pleasure and joy.

*My joy is seeing him well, seeing him happy, seeing him included.* (P8)

*It is very exhausting, it is difficult, but at the same time it is very pleasurable, very rewarding. I do everything with the greatest love in the world. I say that I may not be the best mother in the world, but I do everything to be the best one she could have.* (P12)

*But when we see that she is improving every day, it makes us much happier, and that we are capable of pursuing something more for her, of giving her a better quality of life and for ourselves as well.* (P13)

## DISCUSSION

This study identified the coping modes related to the focal stimulus, which in this case refers to becoming the mother of a child with CZVS, in order to adapt to a new life reality. According to Roy’s Adaptation Model, people are capable of adapting through mechanisms that promote behaviors in response to environmental stimuli^(9)^. In this sense, at different moments of the adaptive process, distinct coping modes in response to the focal stimulus emerged. Initially, in the face of the CZVS diagnosis and without residual stimuli to provide support, the impact of the diagnosis became evident, as well as the feeling of grief. The idealization and life plans for a child usually occur during the gestational period^(15)^. In this regard, having a child with a disability contrasts with the idealized image of the expected child - that is, the expectation of a child with “regular physical and psychological abilities” - triggering feelings such as grief and loss^(5,15)^. The term *adaptation* has been commonly used in Nursing and involves the individual’s adjustment to an illness, disability, or health problem. However, adaptation can be understood as a process, conscious or unconscious, with outcomes of either success or failure, and it encompasses physiological, psychological, and sociocultural mechanisms^(16)^.

It became evident in this study that mothers undergo an adaptation process to meet the complex needs of their children. Caring for children affected by CZVS requires knowledge and skills to deal with episodes of seizures, risk of self-injury, swallowing problems, risk of food bronchoaspiration, insomnia, irritability, and continuous crying without any apparent reason^(4)^. The lack of preparation and knowledge to handle the special care needs of children with CZVS is a reality for many mothers, especially younger and inexperienced ones who are caring for their first child^(4)^. Knowledge about caring for the child may develop empirically, through daily experiences, amid uncertainties, surprises, and shared learning^(5)^. This lived experience may even surpass the guidance provided by health professionals, who sometimes lack skills, have limited knowledge, and offer imprecise information about prognosis^(5)^.

Although such challenges were not observed in the results of the present study, the literature highlights other difficulties faced by these families, such as complex therapeutic itineraries involving multiple outpatient and rehabilitation services, considering emerging complications and the need for multiprofessional care^(6,15)^. Additionally, mothers of children with microcephaly face barriers in accessing specialized services for their children and a lack of psychosocial support for themselves^(2,3)^.

The overload involved in caring for a child with CZVS affected the mothers’ self-care and daily lives. This situation highlights the presence of the physiological adaptive mode, with emphasis on the physical aspects of activity and rest, which are compromised by the dedication required for the child. Having a child affected by CZVS can disrupt or transform the mother’s life plans, routines, and habits^(3,5,6)^. It becomes evident that time is devoted exclusively to caring for the child, to the detriment of the woman’s personal desires, leisure, and self-care, leading to the abandonment of moments of rest and distancing from social activities with friends and family^(5,6)^. Mothers of children with CZVS may also experience overload, exhaustion, lack of time, and the accumulation of other household responsibilities^(5,6)^.

Regarding the overload experienced by mothers of children with CZVS, one study showed a high prevalence of musculoskeletal pain among those whose children had microcephaly resulting from the Zika virus, indicating how much the physiological adaptive mode related to activity was affected. Domains related to physical and emotional health showed the lowest scores, and this pain reduced these women’s quality of life^(17)^. A study conducted in Goiânia, the capital of a state in the Brazilian Midwest, identified factors associated with better quality of life among mothers of children with CZVS: having a partner, engaging in leisure activities, and owning a car as a means of transportation^(18)^. These aspects are recommended for public policies specific to this population and may support different adaptive modes

Regarding the adaptive mode of role performance, many mothers reported feeling an impact on their social roles, as they abandoned formal studies, lost paid employment, and lacked future prospects^(3,6,15)^. Not infrequently, families experience increased financial expenses and difficulties paying their bills, leading them to seek support from non-governmental organizations^(6)^.

A study analyzing access to fundamental social rights among individuals affected by CZVS found that 81% of children using anticonvulsants did not receive these medications from the public health system. Families were also responsible for purchasing nutritional formulas and diapers in 79% and 100% of cases, respectively. Furthermore, 83% of mothers did not return to work after the birth of their children. The study also concluded that these families live at the intersection of providing comprehensive care to a child with special health needs and facing inefficiency and neglect from public authorities, in a context that discourages them and contributes to giving up on seeking their rights^(19)^.

Therefore, the importance of the State’s role in ensuring this well-being is emphasized, in the sense of facilitating not only adaptive modes related to social performance but also the interdependence needed in this context. Furthermore, social accountability must be supported, especially by Nursing, which provides direct care to this population. Roy highlights that nurses possess knowledge because they understand how people respond to their environment^(20)^. Thus, it becomes essential that, upon identifying the needs experienced across the various adaptive modes, nursing - supported by its expertise - helps individuals achieve an adaptive output.

The presence of the self-concept adaptive mode is also notable, expressed through reports related to the mothers’ psychological and emotional aspects. The complexity of the conditions to which they are exposed has repercussions on mental health, leading to the emergence of common mental disorders, depressive/anxious mood, somatic symptoms, decreased vital energy, and depressive thoughts^(5,6)^.

Conversely, in the search for adaptive balance, positive feelings were identified, such as patience, love, willpower, determination, overcoming challenges, adapting to an unexpected situation, solidarity, and resilience^(5)^. However, even so, coping modes can often compromise adaptive processes, especially those related to self-care. A study conducted with mothers of children with CZVS in the state of Sergipe, Brazil, showed that they experience lower levels of personal competence associated with psychological distress and a more fragile social domain of quality of life, as well as lower levels of acceptance of life and of themselves^(21)^.

Therefore, the impact of CZVS on family dynamics and daily routines becomes evident, especially for women^(5)^. Some dedicate themselves to full-time care of the child out of fear that others may not provide adequate care^(5,6)^, as observed in the present study. Low paternal participation was also identified, due to men’s involvement in paid work activities. This situation is closely related to the *interdependence adaptive mode*, in which social and emotional relationships are affected, as well as the performance of social roles themselves^(9)^. There is a lower tendency for men to participate in domestic work and in caring for children with CZVS, generally maintaining the role of providers^(5)^. Gender inequities occur daily and generate care overload for mothers, as well as tensions in marital relationships, including episodes of infidelity, abandonment, and domestic violence^(3)^.

Social support within the family is mostly provided by women from different generations (grandmothers, aunts, sisters)^(5)^. The availability of a social support network has the potential to reduce care overload and mitigate vulnerability to mental health problems^(6)^. Moreover, studies associate fragile social support with greater health inequities, which may reflect difficulties in accessing formal care and support networks^(22)^. In this regard, research conducted with mothers of children with CZVS born in Mato Grosso do Sul, Brazil, revealed important gaps in the care provided to families and children that may affect their quality of life. More than one-third of the women followed in Primary Health Care services did not receive guidance on child follow-up, and 73% were not informed about the availability of social support after birth. Psychosocial support was absent from these women’s therapeutic itineraries^(23)^. In this context, Nursing can play a leading role by facilitating adaptive modes of interdependence, as professionals in this field have the skills to understand people and how they adapt to their environment^(20)^.

The present study showed the use of the internet in shaping support networks. Social media and the internet are considered the main channels for learning through the exchange of information and experiences, an action that promotes well-being and can reduce emotional impact and improve self-care among these mothers^(6)^. In stressful situations, social media can be used not only to search for information but also to obtain social support and emotional encouragement, which helps mitigate difficulties^(24)^. Social media groups can also offer opportunities for interaction among mothers and, therefore, serve as an alternative source of support^(24)^.

### Limitations of the study

This study presents some limitations that deserve consideration. Data collection was conducted remotely, which made it impossible to directly observe the participants’ social and family contexts. This limitation may have restricted a deeper understanding of daily interactions and environmental conditions that influence the maternal adaptation process. Furthermore, the use of social media as the main recruitment strategy may have limited the inclusion of mothers with lower digital access, potentially reducing the socioeconomic diversity of the sample. Despite the geographic reach, the number of participants was small, which limits the generalization of the findings. On the other hand, the virtual format made it possible to capture experiences from different regions of the country, enriching the analysis. It is recommended that future research integrate in-person observation and mixed methods to broaden the understanding of the multiple factors that shape maternal adaptation in the context of congenital Zika virus syndrome.

### Contributions to the field of Nursing, health and public policies

The findings of this study offer relevant contributions to nursing practice by highlighting the modes of maternal adaptation in the face of the challenges imposed by caring for children with congenital Zika virus syndrome. The identification of physical and emotional overload, the reorganization of social roles, and the fragility of support networks signal the need for nursing interventions focused on promoting resilience, providing psychosocial support, and strengthening self-care. In the field of public health, the study broadens the understanding of the vulnerabilities that shape these mothers’ daily lives, emphasizing the importance of multiprofessional and intersectoral action to ensure access to specialized care, social inclusion, and continuous support for families. Regarding public policies, the results reinforce the urgency of strategies that integrate quality care, financial support, and the guarantee of social rights, contributing to reducing inequities and improving the quality of life of mothers and children affected by the syndrome.

## FINAL CONSIDERATIONS

The present study made it possible to understand the process of maternal adaptation to caring for children with CZVS, highlighting both the challenges faced by mothers and the coping mechanisms they adopt. The results showed that adaptation occurs as a process influenced by multiple factors, including the absence of contextual and residual stimuli, care overload, difficulties accessing health services, and the need to reorganize personal and professional life.

Maternal coping unfolds across the adaptive modes described in the theoretical framework adopted for the study. Initially, mothers experience grief over the loss of the idealized child and must develop strategies to deal with the new reality. *Self-concept* is then affected by emotional overload, and significant personal changes are observed in their role performance. *Interdependence* manifests in the search for family support and informal networks that contribute to maternal adjustment and well-being.

The findings of this study contribute to expanding knowledge about the experiences of mothers caring for children with CZVS and reinforce the importance of strengthening intersectoral actions aimed at supporting these women, including access to multidisciplinary care, psychosocial assistance, and - according to recent political discussions - continuous financial support. Additionally, the need to raise awareness among health professionals is emphasized, so that care and reception of these families are improved in ways that promote maternal adaptation and strengthen caregivers in fostering the quality of life of both the children and themselves.

## Data Availability

Research data are availability in the article.
