# Supplementary material for: Maternal adaptation to caring for a child with congenital Zika virus syndrome
Source: Rev Bras Enferm. 2026 Oct 16;79:e20250475. doi: 10.1590/0034-7167-2025-0475 (PMC13679172; doi:10.1590/0034-7167-2025-0475)
Supplement: Translation

# Translation: Adaptação materna aos cuidados da criança com síndrome congênita do Zika vírus

**DOI:** 10.1590/0034-7167-2025-0475pt

**Authors:** Laís Celeste Medeiros Mendes da Fonseca, Fernanda Machado Silva-Rodrigues, Natália Maria Vieira Pereira-Caldeira, Aline Cristiane Cavicchioli Okido, Lucila Castanheira Nascimento, Diene Monique Carlos, Flávia Azevedo Gomes-Sponholz, Nayara Gonçalves Barbosa

**Affiliations:** I Universidade Federal de Juiz de Fora. Juiz de Fora, Minas Gerais, Brasil; II Universidade de São Paulo, Escola de Enfermagem. São Paulo, São Paulo, Brasil; III Universidade de São Paulo, Escola de Enfermagem de Ribeirão Preto. Ribeirão Preto, São Paulo, Brasil

**Keywords:** Transmissão Vertical de Doenças Infecciosas, Síndrome Congênita de Zika, Relações Mãe-Filho, Habilidades de Enfrentamento, Enfermagem.

## Abstract

**Objetivo:**

compreender o processo de adaptação materna aos cuidados da criança com a Síndrome Congênita do Zika vírus.

**Métodos:**

estudo descritivo, qualitativo, fundamentado no Modelo de Adaptação de Roy. Foram entrevistadas, remotamente, 20 mães cuidadoras de crianças com esta síndrome, de diferentes estados brasileiros, no período de janeiro a outubro de 2024. Os dados foram submetidos à análise temática reflexiva.

**Resultados:**

As mães vivenciaram desafios que permearam o diagnóstico, luto do filho idealizado e adaptação à nova realidade. Os modos adaptativos destacaram a sobrecarga física e emocional, a reorganização do autoconceito e do desempenho de papéis, além da fragilidade das redes de apoio.

**Considerações finais:**

Os resultados reforçam a necessidade de apoio profissional e de estratégias intersetoriais que ampliem o suporte às mães, no sentido de promover assistência psicossocial, inclusão social e acesso a cuidados especializados.

## INTRODUÇÃO

A infecção pelo Zika vírus representa uma relevante questão de saúde para as gestantes, pois o vírus pode ser transmitido pela via transplacentária e causar efeitos teratogênicos no feto. O conjunto de espectro de sinais e sintomas desses efeitos foi designado Síndrome Congênita do Zika vírus (SCZV), e inclui uma gama de anormalidades estruturais no cérebro e disfunções nas crianças afetadas, secundárias aos danos no sistema nervoso central^(1,2)^. A SCZV manifesta-se, principalmente, por microcefalia, presente em aproximadamente 80% dos casos. Além disso, podem ocorrer diversas complicações neurológicas, visuais, motoras e auditivas^(1-3)^. A infecção pelo Zika vírus durante o primeiro e o segundo trimestres da gestação apresenta risco aumentado de restrição do crescimento intrauterino e de comprometimento do sistema nervoso central fetal^(1)^.

Na América Latina ocorreu uma epidemia de infecção pelo Zika vírus entre os anos de 2015 e 2019, o que resultou no nascimento de crianças com SCVZ^(4)^. O Brasil foi o país mais acometido, com 80% dos casos^(5)^, e continua a registrar novos casos, em um sinal de constante ameaça à saúde materno-infantil^(6)^. Destaca-se que as populações mais vulneráveis a esta infecção apresentam condições socioeconômicas precárias, baixa escolaridade, habitações inadequadas, saneamento deficiente e alta exposição aos vetores da doença^(3,6)^. Tais populações possuem ainda acesso reduzido aos serviços de saúde reprodutiva, como cuidados pré-natais e aconselhamento contraceptivo, o que assevera as iniquidades em saúde^(1)^. Ademais, as desigualdades sociais e pobreza são ainda mais acentuadas para as mulheres responsáveis pelo cuidado da criança^(3)^.

As repercussões sociais, econômicas, culturais e pessoais desta epidemia permanecem desconhecidas, e sua magnitude representa um desafio para os sistemas de saúde e qualificação dos profissionais^(1)^. As consequências da SCVZ envolvem não somente as crianças acometidas, mas suas famílias, comunidade e cuidadores, sobretudo as mães que apresentam maior tendência ao sofrimento mental dada a complexidade do cuidado requerido pelas crianças^(2)^. As crianças com SCZV podem apresentar limitações de funções físicas ou mentais, dependência medicamentosa, dietética, tecnológica, necessidade de terapia de reabilitação e de cuidado multidisciplinar^(7)^, o que as caracteriza como pertencentes ao grupo de crianças com necessidades de saúde especiais. Tal grupo necessita de maior atenção e acompanhamento dos serviços de saúde em relação a outras crianças na mesma faixa etária^(8)^.

As vivências no cuidado de crianças com SCZV envolvem problemáticas concretas e dilemas cotidianos em relação aos aspectos afetivos, além de reconfiguração dos projetos de vida, sobrecargas física, psicológica e financeira, desafios no acesso aos serviços de saúde e incertezas em relação ao futuro e ao desenvolvimento das crianças. Essas emoções incluem culpa, medo, tristeza, solidão, incerteza, estresse, ansiedade, adaptação, estigma e preconceito social pela condição da criança^(3,5,7)^. Nessa perspectiva, muitas mães vivenciam condições de distanciamento de suas atividades sociais, culturais e de lazer, bem como fragilidade da rede de apoio e necessidade de compartilhamento de cuidados^(6,7)^, com repercussões na saúde e bem-estar materno^(1)^.

Ainda nesse contexto, são expostas a vários estímulos que interferem no estado de adaptação à nova situação que envolve a criança com SCZV. Assim, torna-se necessário um olhar voltado para a adaptação dessas cuidadoras dadas as significativas limitações impostas pela doença e suas representações. Para tanto, o modelo teórico proposto por Callista Roy apresenta-se como uma importante ferramenta, uma vez que, nesta teoria, a pessoa é vislumbrada como um sistema holístico adaptativo, que emite respostas adaptativas ou ineficientes. Ademais, o ambiente é percebido com todas as condições, circunstâncias e influências que envolvem e impactam no desenvolvimento e comportamento da pessoa^(9,10)^. Nessa perspectiva, o cuidado de enfermagem implementado objetiva a promoção de respostas adaptativas da pessoa ^(9,10)^.

O aprofundamento neste tema poderá subsidiar a implementação de estratégias de atenção junto às mães e famílias no cuidado dessas crianças, ao considerar o caminho por elas percorrido diante dos estímulos de vida, ou seja, das consequências da SCZV e do envolvimento como cuidadoras da criança, em busca de desenvolver a capacidade adaptativa^(9)^.

## OBJETIVO

Compreender o processo de adaptação materna aos cuidados da criança com a Síndrome Congênita do Zika vírus.

## MÉTODOS

### Aspectos éticos

O estudo foi conduzido conforme diretrizes éticas nacionais e internacionais e aprovado por Comitê de Ética em Pesquisa, integrante do sistema CEP/Conep, em 30 de setembro de 2022. Para preservar o anonimato das participantes, as entrevistas foram identificadas por código alfanumérico, com a letra ‘P’ (participante), seguido pelo número arábico correspondente à ordem cronológica de realização das entrevistas (P1 a P20).

### Tipo de estudo

Estudo qualitativo, fundamentado no Modelo de Adaptação de Roy (MAR). Para Roy, os indivíduos vivem em constante interação com o meio, ao mesmo tempo em que são expostos a estímulos por ela denominados de *input*. Tais estímulos são classificados em focais, podendo estes ser internos ou externos, contextuais e residuais. A combinação dos estímulos compõe o nível de adaptação do indivíduo, ou seja, a capacidade de enfrentar e reagir às mudanças ao ambiente. A resposta ou reação a esses estímulos pode ser efetiva de adaptação ou ineficaz de desadaptação, denominada de *output* ^(9,10)^.

O MAR é composto por quatro modos adaptativos que são produzidos ou manifestados. No modo fisiológico, o indivíduo reage aos estímulos produzidos pelo ambiente diante das cinco necessidades básicas: oxigenação, nutrição, eliminação, atividade e repouso. O modo autoconceito compreende os aspectos psicológicos, morais e espirituais, juntamente com os valores, crenças e sentimentos do indivíduo. O modo de desempenho é de natureza social e relaciona-se com os papéis assumidos pelo indivíduo na sociedade. Por fim, o modo interdependência, também de natureza social, diz respeito às interações, em dar e receber amor, respeito e valor^(9,10)^.

Dada a complexidade que envolve a adaptação das mães de crianças acometidas pela SCZV, é fundamental analisar quais elementos se fizeram presentes em seus mecanismos de enfrentamento frente a este novo estímulo de ser mãe de uma criança com SCZV, quais os modos adaptativos desenvolveram neste processo e qual/quais os resultados de comportamento (adaptação ou não-adaptação) apresentaram diante desta nova condição de vida. O estudo seguiu as diretrizes do Critério Consolidado de Relato de Pesquisa Qualitativa (COREQ)^(11)^.

### Local, População e Critérios de seleção

Foram incluídas mães biológicas de crianças com diagnóstico de Síndrome Congênita do Zika vírus, maiores de 18 anos, localizadas por intermédio de redes sociais. Excluíram-se mães biológicas que não eram responsáveis pelos cuidados da criança, sendo estes exercidos por outro cuidador responsável, e mães de adolescentes.

### Coleta de dados

Os dados foram obtidos no período de janeiro a outubro de 2024. O recrutamento das potenciais participantes do estudo foi realizado por meio da divulgação da pesquisa nas redes sociais, especificamente Instagram® e Facebook®. Para localizar perfis potencialmente elegíveis, foram utilizados os seguintes unitermos nas buscas: zika vírus, zika e gravidez e síndrome congênita zika vírus. As buscas foram feitas manualmente, por meio das ferramentas de pesquisa interna das plataformas, a fim de identificar publicações, perfis, páginas de organizações não governamentais (ONGs) que apoiassem famílias de crianças com ZCZV e grupos cujos conteúdos se relacionassem com a temática do estudo. Realizou-se uma busca na lista de seguidores dos grupos ou das páginas, com atenção especial às usuárias que interagiam nas postagens.

Preconizou-se a busca por perfis abertos nas redes sociais, os quais, muitas vezes, continham em suas descrições referência a filho(a) com ZCZV, bem como divulgação pública de fotos da criança. Uma vez identificados os perfis (n = 168), enviou-se um convite individual, via mensagem direta nos perfis das participantes cadastrados nas redes sociais. Essa mensagem incluía uma apresentação da pesquisadora principal, os objetivos do estudo e um endereço de e-mail para contato, com o intuito de esclarecer dúvidas e formalizar o interesse na participação.

A técnica de amostragem utilizada foi a bola de neve^(12)^, que consiste em solicitar às participantes iniciais que indiquem outras pessoas com características semelhantes às pretendidas para o estudo. Assim, além de convites diretos, algumas participantes sugeriram outras mães que poderiam ter interesse em contribuir com a pesquisa, ampliando a diversidade de dados de interesse para a pesquisa.

Disponibilizou-se o Termo de Consentimento Livre e Esclarecido para leitura. Após a concordância, as potenciais participantes foram contatadas novamente para agendamento das entrevistas semiestruturadas, realizadas de forma remota e gravadas por meio da plataforma *Google Meet*®. As entrevistas foram conduzidas por uma enfermeira *(iniciais omitidas para avaliação cega por pares)*, especialista em saúde da mulher e da criança, mestranda, com experiência prévia e treinamento específico na condução de entrevistas qualitativas. Elas tiveram duração média de 29 minutos e ocorreram em um único encontro. Ao todo, 21 mães manifestaram interesse em participar, das quais 20 efetivamente participaram do estudo. Houve uma única recusa, motivada pela escolha da participante em não compartilhar questões pessoais.

Foram coletados dados sociodemográficos como: idade materna, cor autodeclarada, escolaridade, estado civil e número de filhos. Quanto aos dados da criança, foram obtidos: idade e sequelas da SCZV. Foram utilizadas as seguintes questões norteadoras: Como foi a descoberta do diagnóstico da síndrome congênita pelo Zika vírus? Quais sentimentos você vivenciou naquele momento? Como você lidou com esses sentimentos? Alguém a apoiou? Quais foram as consequências da doença na criança e quais as necessidades de cuidado com seu filho? Você possui uma rede de ajuda? Como foi a sua adaptação à nova realidade? Como é a sua rotina de cuidados da criança em casa?

A inclusão dos participantes foi encerrada após a 20ª entrevista, quando a análise concomitante dos dados indicou a ocorrência de saturação teórica, definida como o ponto em que as informações se tornaram reincidentes e permitiram aprofundamento das questões de estudo, ancoradas no modelo teórico^(12)^. Para assegurar a observância desse critério, foi empregada a codificação temática, conduzida por dois pesquisadores, que monitoraram a recorrência dos códigos e atestaram a ausência de dados inéditos capazes de contribuir significativamente para a compreensão do fenômeno investigado^(12,13)^.

### Análise dos dados

Os dados obtidos por meio das entrevistas foram analisados de acordo com o referencial da análise temática reflexiva, segundo o proposto por Braun e Clarke^(14)^. Com base nesta modalidade de análise, privilegiou-se ir além do conteúdo semântico dos dados, para examinar ideias, suposições e idealizações dos participantes. Foram percorridas as seis fases previstas: 1) familiarização dos dados, por meio de leituras verticais e horizontais do conjunto de dados; 2) geração dos códigos iniciais, a partir de elementos que respondiam às questões do estudo; 3) busca por temas em que, com base na fase anterior, os códigos foram agrupados à luz do referencial teórico; 4) revisão dos temas; 5) definição e denominação dos temas; e 6) elaboração do relatório^(14)^.

A análise foi conduzida por duas pesquisadoras *(iniciais omitidas para avaliação cega por pares)*, com vistas a assegurar o rigor metodológico e a redução de vieses preconceituais^(14)^. Os códigos obtidos na primeira fase foram checados pelas pesquisadoras seniores *(iniciais omitidas para avaliação cega por pares)* e, após validação, organizados em temas durante reuniões entre membros do grupo de pesquisa. Eventuais divergências entre os autores foram resolvidas nas reuniões, até a obtenção de consenso.

## RESULTADOS

Participaram 20 mães biológicas de crianças com síndrome congênita do Zika vírus, com idades entre 24 e 45 anos. Em relação à raça, 11 se autodeclararam pardas, cinco brancas e quatro pretas. As mães residiam em diferentes estados brasileiros, sendo seis no Ceará, três na Bahia, três na Paraíba, duas no Rio Grande do Norte e uma participante em cada um dos seguintes estados: Pernambuco, Goiás, Tocantins, Sergipe e Rio de Janeiro. A maioria era casada ou vivia em união estável (n=10), tinha ensino médio ou superior completo (n=16) e se dedicava às atividades do lar (n=19). A religião católica foi elencada por 10 mães. O número de filhos variou entre 1 e 4. A idade das crianças com síndrome congênita do Zika vírus foi de 5 a 9 anos, todas com microcefalia, 11 com paralisia cerebral e 10 com comprometimento ocular.

A partir da análise dos dados, foram construídos três temas: i) Do luto pela perda do ideal até a adaptação a uma nova realidade; ii) Demandas de cuidado da criança com Síndrome Congênita do Zika vírus: repercussões maternas pessoais e sociais; iii) Redes de apoio e estratégias de adaptação. Esses temas serão apresentados a seguir.

### Do luto pela perda do ideal até a adaptação a uma nova realidade

Este tema descreve os mecanismos de enfrentamento das mães diante do diagnóstico da SCZV. As participantes relatam que, em um primeiro momento, tais condutas estavam relacionadas ao choque diante do novo, bem como ao desempenho de novos papéis, principalmente o de cuidadora de uma criança com necessidades de saúde especiais. A ausência de estímulos residuais, somada ao ineditismo da experiência de ter um filho com alguma deficiência, fez com que a SCZV, por ser uma condição nova segundo os critérios de diagnóstico e prognóstico, tornasse o processo adaptativo a essa realidade ainda mais complexo, conforme é possível observar abaixo:

*Abriu um buraco debaixo dos meus pés porque, até então, eu já tinha dois filhos. Na minha família não tinha, nunca teve ninguém com necessidade especial… Nunca teve ninguém que tivesse nenhum problema ou má formação. E foi isso que foi, pra mim, uma coisa nova, diferente.* (P7)

*Eu fiquei sem reação, porque é algo novo. É uma deficiência em si, mas é algo novo. Você não tá acostumada a todo dia, ou quando você tá grávida, que a sua criança nasça com alguma deficiência. Foi algo novo, inesperado, que ninguém imaginava. Então, todo mundo ficou muito assustado.* (P13)

*Eu me vi assustada, em pânico porque eu não sabia o que era exatamente isso que tinham me dito e para onde o que ia dar, ao mesmo tempo que eu me senti em luto por não ter nascido a criança que eu esperava.* [...]. (P6)

Outra participante descreveu viver o luto não apenas como um fato isolado, decorrente da perda do filho idealizado, mas como um processo contínuo a cada nova necessidade de saúde da criança. Esse ciclo se perpetuou, em parte pela ausência de estímulos contextuais e residuais que pudessem apoiar outros mecanismos de enfrentamento:

*Eu vivi o luto por um ano, depois eu comecei a lidar melhor. Eu falo lidar, mas, em cada diagnóstico que eu recebo, em cada: “oh, precisa fazer cirurgia”, a gente vai se recompondo. A*t*é certa idade, a gente tinha aquela esperança, mas o tempo foi passando e a gente foi entendendo que não, que tinha que ser daquela forma que a gente precisava ser forte. E foi muito difícil pra gente.* (P18)

Com o diagnóstico da doença, surgiram diversas demandas e necessidades de cuidado da criança - estímulo contextual. No início do processo adaptativo, as mães relataram medo e insegurança quanto à capacidade de cuidarem da criança, especialmente por dificuldades impostas pelas sequelas e consequências da SCVZ. Nesse contexto, observou-se a emergência de comportamentos voltados à articulação de mecanismos relacionados ao autoconceito e ao desempenho de papéis como formas de enfrentamento da nova realidade. Entre os desafios mencionados, destacaram-se dificuldades na alimentação da criança, episódios de crises convulsivas, além de irritabilidade e agitação:

*E aí vem o medo, o receio de não saber cuidar. É o meu primeiro e único filho. Então, além de não saber cuidar direito de um recém-nascido em si, ainda tinha a situação da criança que não estava mamando, que não estava sugando... Era uma criança que dava crise convulsiva, eu não sabia que estava dando crise convulsiva, era uma criança muito irritada e que não dormia à noite. Foi muito complicado.* (P2)

*No começo, era uma bebê normal, que mamou direitinho, até seis meses* [...]. *Conforme ela foi crescendo, foi começando a mudar. Então, o que a gente vê aí, ela começava com dificuldade, a tomar o leite líquido. Aí foi feito vários exames para descobrir se ela se engasgava com o leite líquido ou era com o grosso* [pastoso]. *Então, a gente foi adaptando.* (P13)

### Demandas de cuidado da criança com Síndrome Congênita do Zika vírus: repercussões maternas pessoais e sociais

A partir do processo adaptativo inicial, caracterizado pelo diagnóstico de SCZV, as mulheres relataram o modo autoconceito de enfrentamento, envolvendo a integridade psíquica e espiritual. Nesse sentido, buscaram se adaptar ao novo “eu” e às suas novas realidades. Elencaram, como uma das principais necessidades de cuidados, a administração de dieta via gastrostomia ou trituração dos alimentos, quando da alimentação por via oral, muitas vezes, devido à disfagia. Na rotina de cuidados, destacaram a administração das medicações, oxigenoterapia, trocas de fraldas e banho, contextualizadas no cuidado solitário e na interdependência:

*Administro a dieta. A hidratação, as medicações, troca de fralda, banho, essas coisas.* (P12)

*Ela tem uma rotina de cuidados. Aí sempre tem que ter cuidado para o horário dar o leite na hora certa, deitar, botar na cadeira de rodas. Ela tem oxigênio….* (P20)

[...] *Por exemplo, na hora da alimentação, ele demora muito, na hora de comer, eu prefiro dar ali devagar, do que dar rápido e ele engasgar, broncoaspirar.* (P2)

*Hoje ela depende exclusivamente de mim. Seja pra pegar fralda, seja pra se alimentar, seja pra tomar um banho, pra vestir uma roupa.*(P13)

Os processos de crescimento e desenvolvimento das crianças, geralmente objetos de vigilância constante para a maioria das mães, não apenas geraram expectativas quanto à evolução ao longo do tempo como trouxeram preocupações e demandas progressivas. A dificuldade de comunicação por parte das crianças dificulta a compreensão de suas necessidades específicas, ao mesmo tempo em que processos típicos e esperados do crescimento, como o ganho ponderal, resultaram em novas demandas de cuidado e sobrecarga para as mães:

*Ela não verbalizar é uma dificuldade pra mim. Acho que por isso que eu fico tão intensa com ela 24 horas por dia* [...]. *Eu sou muito preocupada em relação ao cuidado dela. Isso é um dos fatores que psicologicamente me abala muito, sabe?* (P10)

*A maior dificuldade hoje é o peso dele pra mim. Transportar, mudar pra cá, pra lá. É o dia a dia, tá cada vez mais difícil. Porque eu vejo que daqui a três ou quatro anos, eu vou precisar de ajuda para fazer um deslocamento simples* [...]. *Eles crescem, né?* (P8)

Para atender às necessidades dos filhos, algumas mães tiveram que fazer renúncias, como abandonar seus empregos e a vida profissional ou adaptar suas rotinas de trabalho para dedicação integral aos cuidados com a criança. Nesse sentido, frente ao estímulo focal e contextual apresentado, foi possível identificar modos de enfrentamento que impactaram as relações sociais e suas condições prévias como mulheres autônomas - ou seja, um modo interdependente de comportamento, para tornar possível o cuidado do filho:

*Logo que ele nasceu, eu pedi demissão* [...]. *E passei a viver cuidando dele. E ainda hoje eu não me vejo deixando ele com alguém, trabalhando, porque é muito puxado, é muito corrido.* (P8)

*Na verdade, a minha vida parou ali. E hoje eu sou mais a mãe, a cuidadora, do que eu mesma, entendeu? Eu não consegui voltar a trabalhar. Então, assim, meio que abandonei a minha vida e virei só a mãe de Manoela* [nome fictício] *e a cuidadora dela.* (P12)

As múltiplas demandas e a necessidade de conciliar o cuidado contínuo da criança com as demais responsabilidades familiares ocorreram frequentemente às custas de renúncias da vida social, do autocuidado e da própria qualidade de vida das participantes:

*Hoje, com relação à minha vida, a cuidar dela e ser mãe, o sentimento que eu tenho é de muito amor e gratidão. Eu sinto falta, né, de ser eu. Eu sinto falta disso, não vou negar.* (P12)

*Assim, questão de sair para uma festa, de sair com as amigas, eu tenho muita dificuldade, porque eu tenho minha família que me dá apoio, mas todo mundo trabalha, então não é sempre que eu consigo deixar ela com alguém.* (P12)

Neste âmbito, duas participantes relataram modos de enfrentamento para minimizar os impactos no autocuidado pelas demandas com a criança: uma delas levava a filha ao salão de beleza junto com ela, enquanto a outra desenvolveu habilidades próprias para realizar o seu autocuidado. Percebeu-se, então, um modo de desempenho de papel que se articula com o autoconceito para enfrentamento e resposta ao estímulo focal:

*Então eu parei de trabalhar, só trabalhava no pai dela. E hoje eu só cuido de Beatriz* [nome fictício]*… Sempre levando ela pra fazer uma unha, fazer o cabelo com ela.* (P19)

*Eu mesmo faço meu autocuidado, hoje eu consigo. Eu assisto vídeo e eu aprendi a fazer tudo, não vou pro salão.*(P16)

### Redes de apoio e estratégias de adaptação

Este tema desvelou um processo adaptativo para cuidado da criança com SCZV, que busca um modo de interdependência, mas por meio de uma rede de apoio fragilizada. Neste âmbito, os pais (gênero masculino) da criança, quando presentes, direcionam suas atividades para a subsistência da família, que muitas vezes enfrenta dificuldades financeiras. No caso de algumas participantes, o apoio das avós foi uma das alternativas encontradas:

[...] *Só o meu esposo no caso. Quando ele tá em casa, porque ele trabalha.* (P12)

*Eu nunca deixo com ninguém mesmo porque eu não tenho ninguém. O pai dele nunca tem folga.* [...] *Porque é questão de gente pra comer, porque quando a gente planeja ter uma criança, a gente planeja dentro do orçamento.* (P7)

*Sempre foi eu e o meu esposo. O meu apoio sempre foi ele. Não foi de outra pessoa da família, não. Até porque a minha família mora em outra cidade, mais distante.* (P13)

*Às vezes, minha mãe* [avó]*, mas, como ela trabalha, aí fica complicado, sabe? Aí, no final de semana, eu tenho o suporte do pai.* (P15)

Houve também relatos sobre a existência de uma rede informal de apoio, composta por outras mães e famílias de crianças também diagnosticadas com a síndrome, com as quais era possível trocar informações e compartilhar experiências, tanto de forma virtual quanto presencial. Isso proporcionou, de certa forma, apoio emocional:

[...] *E depois foi mesmo a corrente que foi realizada através de famílias, que estava na mesma situação, que a gente foi se conhecendo e se colocar num ambiente que várias pessoas estavam passando pela mesma situação acabou que foi confortando.* (P2)

*Então, teve uma delas* [mãe de criança com SCZV] *que decidiu fazer um grupo no WhatsApp. E ali a gente foi se conhecendo, foi formando uma família e foi passando informação para outra* [...]. (P6)

*Eu fiz muitas amizades, eu conheço quase todas as mães aqui da cidade. Principalmente da microcefalia ou da síndrome do Zika vírus* [...]*. A gente tem um grupo onde a gente troca informações, uma vai ajudando a outra. Por exemplo, eu não sabia como judicializar nada. Hoje eu já consigo judicializar, eu já sei por onde ir.* (P17)

Em relação às adaptações e superações do processo, os relatos indicam resiliência e fortalecimento pessoal com o passar do tempo, o que caracteriza o modo de autoconceito e função de papel, sobretudo pelo ímpeto de proporcionar aos filhos uma melhor qualidade de vida e de assegurar seus direitos:

*Eu acho que eu aprendi muito a buscar, a acreditar muito em mim. Eu não achava que eu tinha a coragem que eu tenho hoje, que ele me mostrou que eu tenho. De sair da minha cidade, recomeçar a minha vida do zero, de voltar a estudar, de correr atrás, de brigar judicialmente por coisas dele, de brigar com a escola, de chamar a TV para fazer barraco. Porque a gente é considerada “barraqueira” por brigar pelos direitos do meu filho. Eu aprendi a não temer a nada.* (P17)

*Eu acho que a vontade que eu tenho de prosperar, de dar uma qualidade de vida melhor ao meu filho, de poder dar um atendimento melhor a ele, de poder voltar também a trabalhar. Eu estou tentando dar um novo sentido à minha vida.* [...] *Porque eu não quero mais isso do luto. Eu fiz a minha luta.* (P2)

Por fim, apesar de toda repercussão vivenciada, as falas revelam que essas mães foram encontrando o equilíbrio a partir de diferentes modos de adaptação, expresso pela sensação de prazer e alegria:

*A minha alegria é vê-lo bem, é vê-lo feliz, é vê-lo incluído.* (P8)

*É bastante cansativo, é difícil, mas, ao mesmo tempo, é muito prazeroso, muito gratificante. Eu faço tudo com o maior amor do mundo. Eu falo assim, que eu posso não ser a melhor mãe do mundo, mas eu faço de tudo para ser a melhor que ela poderia ter.* (P12)

*Mas quando a gente vê que ela está evoluindo a cada dia nos deixa muito mais felizes e que a gente está sendo capaz de correr atrás de algo a mais pra ela, de dar uma qualidade de vida melhor para ela e para gente.* (P13)

## DISCUSSÃO

Este estudo vislumbrou os modos de enfrentamento do estímulo focal que estão conectados, neste caso, a tornar-se mãe de uma criança com SCZV, para adaptação a uma nova realidade de vida. De acordo com o Modelo de Adaptação de Roy, as pessoas são capazes de se adaptar por meio de mecanismos que favorecem comportamentos em resposta aos estímulos ambientais^(9)^. Nesse sentido, nos diferentes momentos do processo adaptativo, distintos modos de enfrentamento ao estímulo focal se fizeram presentes. Inicialmente, frente ao diagnóstico de SCZV, sem estímulos residuais para apoio, evidenciou-se o impacto do diagnóstico, bem como o sentimento de luto. A idealização e os planos de vida de uma criança usualmente ocorrem durante o período gestacional^(15)^. Nesse sentido, a criança com deficiência contrasta com a idealização do filho esperado, ou seja, com a perspectiva de uma criança com “habilidades físicas e psíquicas regulares”, acionando sentimentos como luto e perda^(5,15)^. O termo adaptação tem sido comumente utilizado na Enfermagem e envolve a adaptação do indivíduo a uma doença, deficiência ou problema de saúde. Contudo, a adaptação pode ser vislumbrada como um processo, consciente ou inconsciente, com desfecho de sucesso ou de fracasso, que perpassa mecanismos fisiológicos, psicológicos e socioculturais^(16)^.

Evidenciou-se neste estudo o processo de adaptação materna para atenção às complexas necessidades da criança. O cuidado com crianças acometidas pela SCZV requer conhecimentos e habilidades diante de episódios de convulsão, risco de autoagressão, problemas de deglutição, risco de broncoaspiração de alimentos, insônia, irritabilidade e choro contínuo da criança sem nenhuma razão aparente^(4)^. O despreparo e a falta de conhecimento para lidar com as necessidades especiais de cuidados com crianças com SCZV constituem uma realidade para muitas mães, sobretudo para aquelas mais jovens, inexperientes, e que lidam com o cuidado do primeiro filho^(4)^. O conhecimento acerca do cuidado da criança pode ocorrer empiricamente, por meio das vivências no cotidiano, em meio à incertezas, surpresas e aprendizados compartilhados^(5)^. Essa vivência pode, inclusive, superar as orientações dos profissionais de saúde que, por vezes, apresentam falta de habilidade, conhecimento incipiente e informações imprecisas sobre prognóstico^(5)^.

Embora não tenha sido observada tal problemática nos resultados do presente estudo, a literatura apresenta outros desafios para essas famílias, como os itinerários terapêuticos complexos com a criança, com atendimentos em diferentes serviços ambulatoriais e de reabilitação, considerando as complicações emergentes e a necessidade do cuidado multiprofissional^(6,15)^. Ainda, as mães de crianças com microcefalia enfrentam barreiras para acesso a serviços especializados para as crianças e ausência de suporte psicossocial para elas próprias^(2,3)^.

A sobrecarga no cuidado da criança com SCZV interferiu no autocuidado e na vida das mães. Tal situação evidencia a presença do modo adaptativo fisiológico com ênfase nos aspectos físicos de atividade e repouso comprometidos pela dedicação às crianças. O acometimento da criança pela SCZV pode promover a ruptura ou transformação dos projetos de vida da mãe, mudanças na rotina e hábitos^(3,5,6)^. Evidencia-se que o tempo é exclusivamente dedicado ao cuidado da criança, em detrimento dos desejos pessoais da mulher, lazer, autocuidado, com o abandono de momentos de descanso e repouso e afastamento das atividades sociais com amigos e familiares^(5,6)^. As mães de crianças com SCZV podem ainda apresentar sobrecarga, exaustão, falta de tempo, além de sobreposição com os demais afazeres domésticos^(5,6)^.

Em se tratando de sobrecarga de mães com crianças com SCZV, estudo mostrou alta prevalência de dor musculoesquelética naquelas cujos filhos apresentavam microcefalia decorrente do Zika vírus, em um sinal do quanto o modo adaptativo fisiológico, relacionado às atividades, foi impactado. Domínios referentes à saúde física e emocional apresentaram os piores escores, e esta dor reduziu a qualidade de vida destas mulheres^(17)^. Estudo desenvolvido em Goiânia, capital de estado do Centro-Oeste brasileiro, identificou fatores associados à maior qualidade de vida de mulheres mães de crianças com SCZV: ter uma parceria, realizar atividades de lazer e possuir um carro como meio de transporte^(18)^. Tais aspectos são recomendados para políticas públicas específicas a esta população e podem apoiar diferentes modos adaptativos.

No que se refere ao modo adaptativo de desempenho de papel, muitas mães demonstraram sentir, por meio de seus relatos, impacto nos seus papéis sociais, uma vez que abandonaram os estudos regulares, perderam vínculo de trabalho remunerado e careciam de perspectivas futuras^(3,6,15)^. Não raro, famílias experienciam aumento dos gastos financeiros e dificuldades de pagar as despesas, com necessidade de buscar apoio em organizações não governamentais^(6)^.

Estudo analisou o acesso de vítimas da SCZV a direitos sociais fundamentais e concluiu que 81% das crianças usuárias de anticonvulsivantes não recebiam esses medicamentos do setor público, além de suas famílias serem responsáveis por comprar fórmulas nutricionais e fraldas em 79% e 100% dos casos, respectivamente. Além disso, 83% das mães não retornaram ao trabalho depois do nascimento de seus filhos. O estudo concluiu ainda que essas famílias vivem na intersecção entre o cuidado integral de uma criança com necessidades de saúde especiais e a ineficiência e omissão de autoridades públicas, em um contexto que as desencoraja e contribui para que desistam de buscar seus direitos^(19)^.

Ressalta-se, portanto, a importância do papel do Estado na garantia deste bem-estar, no sentido de facilitar não apenas modos adaptativos relacionados ao desempenho social como também a interdependência necessária neste contexto. Ademais, o controle social deve ser apoiado, em especial pela Enfermagem, que realiza o cuidado direto a esta população. Roy destaca que os enfermeiros são detentores de conhecimento porque compreendem como as pessoas respondem ao seu ambiente^(20)^. Portanto, torna-se fundamental que, ao identificar as necessidades vivenciadas em seus diversos modos adaptativos, a enfermagem, apoiada em seus conhecimentos, auxilie os indivíduos a alcançarem o *output* adaptativo.

Destaca-se ainda a presença do modo adaptativo de autoconceito verbalizado por meio de relatos relacionados aos aspectos psicológicos e sentimentais das mães. A complexidade das condições às quais são expostas apresenta repercussões na saúde mental, com consequente surgimento de transtornos mentais comuns, humor depressivo/ansioso, sintomas somáticos, decréscimo da energia vital e pensamentos depressivos^(5,6)^.

Em contrapartida, na busca por um equilíbrio adaptativo, foram elencados sentimentos positivos como paciência, amor, força de vontade, determinação, superação, adaptação a uma situação inesperada, solidariedade e resiliência^(5)^. Contudo, ainda assim, os modos de enfrentamento podem, com frequência, comprometer processos adaptativos, em especial no que se relaciona ao autocuidado. Estudo desenvolvido com mães de crianças com SCZV do estado de Sergipe, Brasil, mostrou que elas vivem menores níveis de competências pessoais associados ao sofrimento psíquico e qualidade de vida no seu domínio social mais frágil, e apresentam menores níveis de aceitação da vida e de si mesmas^(21)^.

Evidencia-se, portanto, o impacto da SCZV na dinâmica e rotina familiar, sobretudo para as mulheres^(5)^. Algumas dedicam cuidado à criança em tempo integral, por receio de que outras pessoas não cuidem bem^(5,6)^, conforme evidenciado no presente estudo. Detectou-se ainda baixa participação paterna, pelo envolvimento do homem nas atividades laborais. Tal situação está intimamente relacionada ao modo adaptativo de interdependência, no qual as relações sociais de afeto são influenciadas, além do próprio desempenho de papel social^(9)^. Observa-se menor propensão da participação masculina no trabalho doméstico no cuidado de crianças com SCVZ, mantendo-se, em geral, na função de provedores^(5)^. As iniquidades de gênero ocorrem cotidianamente e geram sobrecarga do cuidado para as mães, bem como tensionamentos nas relações maritais, com episódios de infidelidade, abandono e violência doméstica^(3)^.

O suporte social no âmbito familiar é majoritariamente compartilhado por mulheres de diferentes gerações na família (avós, tias, irmãs)^(5)^. A disponibilidade de uma rede de apoio social tem o potencial de reduzir a sobrecarga do cuidado e atenuar a vulnerabilidade a problemas de saúde mental^(6)^. Ademais, estudos associam o frágil suporte social a maiores iniquidades em saúde, aspecto que pode mostrar a dificuldade de acesso a redes formais de cuidado e apoio^(22)^. Nesse sentido, pesquisa desenvolvida com mães de crianças com SCZV nascidas no Mato Grosso do Sul, Brasil, mostrou importantes lacunas no cuidado às famílias e crianças que podem impactar na qualidade de vida desses indivíduos. Mais de um terço das mulheres acompanhadas em serviços de Atenção Primária à Saúde não receberam orientações sobre o acompanhamento das crianças, e 73% das mulheres não foram orientadas sobre a disponibilidade de apoio social após o nascimento. O apoio psicossocial não se fez presente nos itinerários terapêuticos dessas mulheres^(23)^. Neste âmbito, a Enfermagem pode ocupar lugar como protagonista, ao facilitar modos adaptativos de interdependência, uma vez que o profissional desta área dispõe de habilidade para compreender as pessoas e as formas como elas se adaptam ao ambiente^(20)^.

Evidenciou-se, no presente estudo, o uso da internet na conformação de redes de apoio. As redes sociais e a internet são consideradas os principais canais para aprendizagem através da troca de informações e vivências, ação que promove bem-estar e pode diminuir o impacto emocional e melhorar o autocuidado entre elas^(6)^. Em situações estressantes, as mídias sociais podem ser utilizadas não apenas para a busca de informações, mas também para obter suporte social e apoio emocional, o que contribui para mitigar dificuldades^(24)^. Os grupos de mídias sociais podem ainda oferecer oportunidades de interação entre as mães e, portanto, configurarem-se como um recurso alternativo de suporte^(24)^.

### Limitações do Estudo

Este estudo apresenta algumas limitações que merecem consideração. A coleta de dados foi realizada de forma remota, o que impossibilitou a observação direta do contexto social e familiar das participantes. Essa limitação pode ter restringido a compreensão mais aprofundada das interações cotidianas e das condições ambientais que influenciam o processo de adaptação materna. Ademais, a utilização das redes sociais como principal estratégia de recrutamento pode ter limitado a inclusão de mães com menor acesso digital, potencialmente restringindo a diversidade socioeconômica da amostra. Apesar da abrangência geográfica, o número de participantes foi reduzido, o que limita a generalização dos achados. Por outro lado, a modalidade virtual permitiu captar experiências de diferentes regiões do país, enriquecendo a análise. Recomenda-se que futuras pesquisas integrem observação presencial e métodos mistos, ampliando a compreensão sobre os múltiplos fatores que condicionam a adaptação materna frente à síndrome congênita do Zika vírus.

### Contribuições para a área da enfermagem, saúde e políticas públicas

Os achados deste estudo oferecem subsídios relevantes para a prática da enfermagem ao evidenciarem os modos de adaptação materna diante dos desafios impostos pelo cuidado de crianças com síndrome congênita do Zika vírus. A identificação de sobrecarga física e emocional, reorganização de papéis sociais e fragilidade das redes de apoio sinalizam a necessidade de intervenções de enfermagem centradas na promoção da resiliência, no suporte psicossocial, e no fortalecimento do autocuidado. No âmbito da saúde coletiva, o estudo amplia a compreensão sobre as vulnerabilidades que atravessam o cotidiano dessas mães, destacando a importância da atuação multiprofissional e intersetorial para garantir acesso a cuidados especializados, inclusão social e apoio contínuo às famílias. Em relação às políticas públicas, os resultados reforçam a urgência de estratégias que integrem assistência de qualidade, suporte financeiro e garantia de direitos sociais, contribuindo para reduzir iniquidades e favorecer a qualidade de vida de mães e crianças afetadas pela síndrome.

## CONSIDERAÇÕES FINAIS

O presente estudo permitiu compreender o processo de adaptação materna aos cuidados de crianças com SCZV, evidenciando os desafios enfrentados pelas mães, bem como os mecanismos de enfrentamento por elas adotados. Os resultados demonstraram que a adaptação ocorre de forma processual, sendo influenciada por múltiplos fatores, inclusive por ausência de estímulos contextuais e residuais, sobrecarga de cuidados, dificuldades de acesso aos serviços de saúde e necessidade de reorganização da vida pessoal e profissional.

O enfrentamento materno perpassa os modos adaptativos descritos no referencial adotado para o estudo. Inicialmente, as mães vivenciam o luto pela perda do filho idealizado e necessitam desenvolver estratégias para lidar com a nova realidade. O autoconceito é, então, impactado pela sobrecarga emocional, e nota-se o desempenho de papéis sobre mudanças de ordem pessoal bastante significativas. A interdependência se manifesta na busca por apoio familiar e em redes informais que contribuem para o ajuste e bem-estar materno.

Os achados deste estudo contribuem para a ampliação do conhecimento sobre as vivências das mães no cuidado de crianças com SCZV, e reforçam a importância do fortalecimento de ações intersetoriais voltadas ao suporte dessas mulheres, incluindo o acesso a cuidados multidisciplinares, assistência psicossocial e, conforme discussões recentes no cenário político, apoio financeiro de caráter contínuo. Além disso, destaca-se a necessidade de sensibilização dos profissionais de saúde para a qualificação do atendimento e acolhimento dessas famílias no sentido de promover a adaptação materna e fortalecer os cuidadores para promoção da qualidade de vida das crianças e deles próprios.

## Data Availability

Os dados de pesquisa disponíveis estão no corpo do artigo.
